# Access to support during childbirth?: women’s preferences and experiences of support person integration in a cross-sectional facility-based survey

**DOI:** 10.1186/s12884-023-05962-2

**Published:** 2023-09-16

**Authors:** Michelle K. Nakphong, Patience A. Afulani, James Opot, May Sudhinaraset

**Affiliations:** 1 Department of Medicine, Division of Prevention Science, University of California, San Francisco, San Francisco, CA USA; 2 Department of Biostatistics and Epidemiology, University of California, San Francisco, San Francisco, CA USA; 3 Innovations for Poverty Action, Nairobi, Kenya; 4 Department of Community Health Sciences, Fielding School of Public Health, University of California, Los Angeles, Los Angeles, CA USA

## Abstract

**Background:**

Integrating support persons into maternity care, such as making them feel welcome or providing them with information, is positioned to increase support for women and improve birth outcomes. Little quantitative research has examined what support women need and how the healthcare system currently facilitates support for women. We introduce the *Person-Centered Integration of Support Persons (PC-ISP)* concept, based on a review of the literature and propose four PC-ISP domains—*Welcoming environment, Decision-making support, Provision of information and education* and *Ability to ask questions and express concern*s. We report on women’s preferences and experiences of PC-ISP.

**Methods:**

We developed PC-ISP measures based on the literature and applied these in a facility-based survey with 1,138 women after childbirth in six health facilities in Nairobi and Kiambu counties in Kenya from September 2019 to January 2020.

**Results:**

We found an unmet need for integrating support persons during childbirth. Between 73.6 and 93.6% of women preferred integration of support persons during maternity care, but only 45.3–77.9% reported to have experienced integration. Women who reported having a male partner support person reported more PC-ISP experiences (*B*0.13; 95% CI 0.02, 0.23) than those without. Employed women were more likely to report having the opportunity to consult support persons on decisions (aOR1.26; 95% CI 1.07, 1.50) and report that providers asked if support persons should be informed about their condition and care (aOR1.29; 95% CI 1.07, 1.55). Women with more providers attending birth were more likely to report opportunities to consult support persons on decisions (aOR1.53; 95% CI 1.09, 2.15) and that support persons were welcome to ask questions (aOR1.84, 95% CI 1.07, 2.54).

**Conclusions:**

Greater efforts to integrate support persons for specific roles, including decision-making support, bridging communication and advocacy, are needed to meet women’s needs for support in maternity care.

**Supplementary Information:**

The online version contains supplementary material available at 10.1186/s12884-023-05962-2.

## Background

Access to support persons has implications for the quality of maternity care and birth outcomes. Lack of support is associated with higher mistreatment during maternity care in facilities, lower person-centered maternity care, higher likelihood of adverse birth outcomes and worse postpartum mental health [1–6]. Yet, the majority of women globally lack access to the support they need during facility-based childbirth [7]. In Kenya, studies across settings indicate that women’s access to a support person is low; in one study, less than half were allowed a companion during labor and only one in five during childbirth [8, 9]. A systematic review of companionship found that even when support persons are allowed to stay with women during maternity care, they are not well integrated into the care system [10]. Provider and facility barriers (e.g., negative provider attitudes, unevenly enforced policies, lack of resources) inhibit support persons’ abilities to effectively provide support [7, 11]. Integrating support persons into maternity care, such as making them feel welcome or providing them with information, has been proposed to increase support for women in facility-based childbirth and improve birth outcomes [10].

We must address critical gaps in understanding what support women need during childbirth and how the health care system currently facilitates women’s support of choice to effectively design interventions to increase support for women. Literature has been dominated by research on labor and birth companionship, but up to 60% of women in some studies in Kenya did not want birth companions [8, 12]. Instead, evidence suggests that women want their support persons integrated into maternity care in other ways, such as bridging communication with providers and to provide consult on decisions [13, 14]. Literature has also focused on male partner involvement, but many women do not want their male partners as birth companions, preferring other types of support persons, such as mothers or sisters [12]. A more granular assessment of women’s preferences for support, including the roles that support persons play and a broad array of support person types, is needed to design maternity care systems that ensure women have the support they really need.

How the health care system currently interacts with and facilitates supportive functions for women beyond labor and birth companionship, such as decision-making support, needs to be examined. The few studies that surveyed women about how the maternity care system facilitated support were qualitative studies [1, 10, 15]. We lack, however, quantitative data about how and to what extent the health care system integrates support persons in practice.

Better understanding is needed of the multi-level determinants that shape women’s preferences to support persons. Few studies have explored the factors that contribute to women’s preferences for support including types of support persons and the kind of support they need. Evidence also indicates that women in Kenya may have different access to support persons based on social status, health condition or support persons’ characteristics [8, 9]. In addition, qualitative studies have emphasized how provider practices, facility policies, infrastructure and level of resources influence women’s preferences for support, as well as how support persons are excluded or integrated into the model of care [7, 16–19]. Efforts to facilitate support for women must be made at multiple levels, from individual providers to organizations and systems [20].

To address these gaps, we developed the concept of *Person-Centered Integration of Support Persons (PC-ISP)* based on a review of the literature. We define PC-ISP as the extent to which support persons are integrated into maternity care that is respectful and responsive to women’s needs and preferences, which include clinical decisions [21]. We also broadly define support persons as lay persons (i.e., those who are not medical professionals employed by the facility) who accompany women, in or near the maternity ward during labor, childbirth or postpartum [18, 22]. Doulas, Accredited Social Health Advocates (ASHAs) or community health workers who provide support may also be included within this definition. We did not use the term “birth companions” in this study because they have been specifically defined as support persons who are present with women during labor and/or childbirth and we instead sought to investigate the ways women may look to a broader definition of support person throughout all maternity care [7, 10]. Integration refers to the process of incorporating support persons into the health care system and the women’s support team [10].

Using the PC-ISP concept, main objectives of this study were to quantitatively examine women’s (1) preferences for support and (2) experiences of whether support persons were integrated into maternity care. Using a socio-ecological approach, we also investigated how factors at women’s, support person’s, household and facility levels were associated with both women’s PC-ISP preferences and experiences.

## Methods

This study used women’s survey data from the Strengthening Person-centered, Accessibility, Respectful Care and Quality (SPARQ) study. Data were collected between September 2019 and January 2020 from women who recently gave birth and their support persons in six facilities in Kiambu and Nairobi counties. These were selected in a mix of public and private facilities (three public hospitals, two private hospitals, one public health centre) with high patient volumes, ranging from 100 to 900 births per month.

Inclusion criteria for women were (a) between 15 and 49 years of age, (b) spoke English or Kiswahili, (c) had given birth vaginally and (d) owned a mobile phone and (e) felt comfortable being contacted by the study team. We excluded women who had cesarean births, because the experience of care and support varies widely from those who gave birth vaginally, especially because cesarean births may arise from emergency conditions. Women who have cesarean births may have unique needs or different preferences for support since their care is more invasive, births may be more complicated and recovery more challenging. Women were recruited from postpartum wards within 48 h of childbirth by female research assistants in collaboration with facility staff. Researchers worked closely with facility managers to secure a private room or space that was located far from the maternity department to ensure privacy. Facility staff approached women who met eligibility criteria and asked if they would be interested in the study. Women were then referred to research assistants, leading them to a private, confidential setting within the facility. To reduce desirability bias, we ensured that providers did not know women’s interview responses. Research assistants described the study, assessed eligibility, obtained informed consent and interviewed the women. Women were reassured that their responses would be kept confidential, that the facility would not know what they had said and that the researchers were not affiliated with the facility. Research staff were trained to allow the mothers to nurse and care for their newborns at any point during the interview and allowed women to take breaks at any point. Consideration was given to women who had recently given birth as vulnerable participants with the possibility of experiencing health problems while participating. If any participant faced health issues during any study-related activity, a referral procedure was in place to ensure that women received immediate care in the facility. Study staff made sure to fully inform the woman’s nearest family member or next of kin about any health problem if they were not present at that time. Interviews lasted approximately one hour and participants received compensation of approximately $1.00 USD worth of airtime sent to their phones as a token of appreciation for their time. A total of 1,197 women provided baseline data and we excluded 59 women who did not report a support person. The final sample included 1,138 women who reported that they had someone either accompany them to the facility or stay with them during labor, childbirth or postpartum.

### PC-ISP measures

To initiate the process of operationalizing PC-ISP, we conducted a literature review of women’s and support persons’ experiences in maternity care and identified themes regarding how support persons were excluded from maternity care practices, such as making them feel unwelcome or failure to communicate with them [23, 24]. We used these themes to specifically define four corresponding sub-constructs of PC-ISP: *Welcoming environment, Decision-making support, Provision of information and education* and *Ability to ask questions and express concern*s.

A *welcoming environment* highlights the importance of positive interpersonal relationships between providers and women’s preferred support persons [24–26]. Adequate *decision-making support* promotes women’s autonomy and agency in their own care to consult with support persons about clinical decisions [27–29]. *Provision of information and education* facilitates support persons’ involvement in care and clarifies their roles [16, 30–32]. The *ability to ask questions and express concerns* acknowledges the importance of engagement with providers during care, especially as an avenue to advocate on behalf of the supported women [13, 16, 23, 33].

In addition, we grounded the concept of PC-ISP in person-centered care which asserts that women should be at the center of their own care [15]. Existing models of medical care in low-resource settings often center around the institutions and providers [11]. Practically, person-centered care gives women a voice and acknowledges their needs for support [34, 35]. Although experiences and perceptions of family and community members are also important, evaluating women’s experiences of care from their own perspectives is critical [26]. We therefore must understand PC-ISP as reported by women, measuring how they perceived that support persons were integrated into their care.

The four themes were used to develop PC-ISP questions in the women’s survey. These were designed to measure the extent to which care integrates support persons (in this context, family members). That implies care that is respectful and responsive to women’s needs and preferences (Table 1). Women were surveyed regarding their preferences for PC-ISP using four questions corresponding to three sub-constructs. Women also responded to five questions regarding the experience of how their support persons were (or were not) integrated into care corresponding to four sub-constructs. Possible response options for all PC-ISP survey questions used a 3-point Likert-type scale: agree, somewhat agree and disagree.

For analyses, we conservatively recoded PC-ISP indicators with “Don’t know” responses as “agree,” assuming that support persons were treated positively and excluded answers of “N/A.” Since there were few “somewhat agree” responses (ranging from 1.5 to 3.9%), each measure was recoded as a dichotomous variable (agree + somewhat agree vs. disagree). Two total score measures were constructed for PC-ISP preferences and experiences by summing the count of “agree/somewhat agree” responses across indicators. PC-ISP preference scores (range 0–4) showed questionable reliability (Cronbach’s α = 0.651) and PC-ISP experience scores (range 0–5) showed poor reliability (α = 0.592), indicating a fair amount of multidimensionality among the few indicators [36]. One PC-ISP experience indicator in particular, *felt welcome*, showed low item-rest correlation (0.1820), meaning that the item was not well correlated with total score. Because of this, we omitted *felt welcome* from the summative PC-ISP experience score (range 0–4), resulting in some improvement in reliability (α = 0.616). PC-ISP preference indicators displayed small to moderate correlations (r = 0.20–0.64) while PC-ISP experience indicators showed no to moderate correlations (r = 0.04–0.61).

**Table 1:** Women’s PC-ISP indicators

Women’s PC-ISP indicators				
Subconstruct	Women’s preferences for PC-ISP		Women’s experiences of PC-ISP	
	Indicator Name	Question	Indicator Name	Question
***Welcoming environment***		---	*Felt welcome*	My family member(s) felt welcome by the facility during my childbirth
***Decision-making support***	*Consult decisions*	I wanted to consult my family about decisions about my care for birth	*Opportunity to consult*	I was given the opportunity by my health provider to consult my family about my health care decisions
***Provision of information and education***	*Know condition/care*	I wanted my family to know about my condition/care	*Told condition/care*	I was asked by my health provider if my family should be told about my condition/care
	*Understand condition/care*	I wanted my family to understand my condition/care		
***Ability to ask questions and express concerns***	*Respects values*	I would have liked my family members to make sure my provider respects my values and choices	*Welcome to ask questions*	My family was welcome to ask my health care provider questions
			*Listened to concerns*	My health care provider listened to my family members’ concerns

Women’s individual-level factors included age (continuous), marital status (married or partnered vs. not), parity (primiparous vs. multiparous), educational attainment (primary or less; vocational/secondary; college/university), current employment status (employed vs. not), birthplace (born in Nairobi or Kiambu counties vs. not) and health insurance status (covered under health scheme/insurance vs. not). Given that health status and health conditions could also influence how providers acknowledge women’s preferences and whether they integrate support persons, we also examined self-reported health status (excellent/very good, good, fair, poor/very poor) and women’s reports of birth complications (yes vs. no).

Support Person variables included the total number of support persons reported (continuous) and the types of support persons by their relation to the woman including eight types: male partner, mother, mother-in-law, sister, brother, father (of the woman), other family member and friend/neighbor/other. No woman reported community health workers or doulas. Binary indicator variables were constructed for each of the eight support person types (e.g., male partner support person vs. no male partner support person, mother support person vs. no mother support person). Women also reported on the timing of support, indicating whether support person(s) was/were present with them during different periods of maternity care (e.g., accompanied to the facility, during labor and childbirth, postpartum).

Because a woman’s position within the household influences her expectations and preferences for care, we also examined household factors including household size (continuous) and indicators of women’s empowerment. Women who were married or partnered were asked four questions regarding decision-making power for various household decisions, including woman’s health care, major household purchases, daily household purchases and visits to family or relatives [37]. We constructed a composite variable *Empowered in household decisions*, indicating whether a woman reported involvement in all four types of household decisions (i.e., “woman only” or “jointly”) versus lack of involvement (i.e., “partner only” or “someone else”) in at least one type of household decision [38]. Among women not married or partnered, those aged ≥20 years or the sole adult in their household were coded as being involved in all four types of decisions and we considered adolescent women < 20 years who resided with other adults as not involved in all decisions. Facility factors included type of facility (public hospital; public health centre; private facility), total number of providers who assisted childbirth (continuous) and type of provider during childbirth (doctor or clinical officer, nurse or midwife, other, none).

### Analysis

We examined bivariate associations between dependent PC-ISP measures and all factors at the women’s, support persons’, household and facility levels, using chi-square tests and t-tests. We also assessed: (1) factors associated with individual PC-ISP indicators using multivariable binomial logistic regression and (2) factors associated with the combined PC-ISP scores, using multiple linear regression.

Both sets of analyses followed a sequential, blocked model building approach to examine how each block accounted for variation, beginning with a model including women’s individual factors, then adding increasingly distal levels in each subsequent model (i.e., adding support persons’ factors, then household, etc.) according to the socioecological model [39]. Within each block of variables at a given level, we included theoretically relevant variables (e.g., age, parity) as well as all variables that showed statistically significant (two-sided alpha = 0.05) bivariate associations. Final models included age, parity, education, marital status, birthplace, health insurance coverage and health status (woman-level), number of support persons, types of support persons, timing of support (support person-level), women’s empowerment in the household (household-level), type of facility and number of providers during childbirth (facility-level). We used linktests and Hosmer-Lemeshow goodness-of-fit tests and found no evidence of specification error or poor fit. We did not find any evidence of multicollinearity between factors (VIF = 1.41). We also examined potential outliers using standardized Pearson residuals, deviance residuals and Pregibon leverage. We performed sensitivity analyses excluding potentially influential observations but found that estimates and confidence intervals of associations were minimally affected. We also conducted sensitivity analyses using different constructions of the total PC-ISP score, such as by assigning “somewhat agree” responses a value of 1 and “agree” a value of 2 with consistent results.

We used two methods to account for clustering by facility: First, we constructed single-level regression models with cluster-robust standard errors. Second, we conducted sensitivity analyses by using multi-level models, adding random effects for facilities. Because intraclass correlations were low (< 0.035), we only present results from simple linear regression models. We were also concerned with confounding that may be due to women’s selection of facilities and thus controlled for (1) whether women were referred to a particular facility and (2) whether women reported selection of the facility because of quality of care (e.g., cleanliness, higher quality, more privacy, trusted providers). To assess the extent to which PC-ISP occurred within and across facilities, we also examined facilities separately to explore possible differences in associations between PC-ISP and risk factors by facility.

## Results

Characteristics of the 1,138 women who had a support person accompanying them to the facility or stay in the facility during labor, childbirth or postpartum are described in Table 2. Women reported an average of 1.5 (SD ± 0.7) support persons which included their male partners (59.2%), sisters (16.4%), mothers (8.5%), other family members (20.9%) and friends/neighbors/others (34.3%) (Table 2). Almost all women reported they were accompanied to the facility (94.6%), while only a fraction reported that a support person (or support persons) stayed with them during labor and/or childbirth (7.4%) or during the immediate postpartum period (43.7%). Of the remaining 1,054 women, 317 (27.9% of the fully sampled) women reported wanting a support person during labor and/or childbirth.

**Table 2:** Descriptive characteristics of the 1138 women

Variable	N or mean	% or (SD)
Total participants	1,138	
Age		
Mean age	25.4	(± 5.0)
Parity		
Mean parity	2.0	(± 1.0)
Primiparous	435	38.2%
Multiparous	703	61.8%
Currently married or partnered		
No	200	17.6%
Yes	938	82.4%
Educational attainment		
Primary or less	504	44.3%
Vocational/Secondary	454	39.9%
College/University	180	15.8%
Religion		
Christian	1175	98.2%
Muslim/other	22	1.8%
Currently employed		
No	687	60.4%
Yes	451	39.6%
Birthplace		
Born in Nairobi or Kiambu counties	239	21.0%
Born elsewhere	899	79.0%
Self-rated health status		
Excellent or very good	398	35.0%
Good	456	40.1%
Fair	181	15.9%
Poor or very poor	103	9.1%
Complications during childbirth		
No	1063	93.4%
Yes	75	6.6%
Health insurance/scheme coverage		
Not covered	169	14.9%
Covered	969	85.1%
Support person type*		
Male Partner	674	59.2%
Mother	97	8.5%
Mother-in-law	37	3.3%
Sister	187	16.4%
Father	10	0.9%
Brother	18	1.6%
Other family member	238	20.9%
Friend/neighbor/other	390	34.3%
Total number of support persons		
Mean (min 1- max 6)	1.5	(± 0.7)
Timing of support*		
Accompanied to facility	1,076	94.6%
Labor and/or childbirth	84	7.4%
Postpartum	497	43.7%
Household size		
Mean	4.2	(± 1.4)
Empowered in household decisions		
Involved in some or no decisions	572	50.2%
Involved in all decisions	566	49.7%
Facility type		
Public hospital	834	73.3%
Public Health Centre	137	12.0%
Private facility	167	14.7%
Providers assisting childbirth*		
Mean total number of birth attendants	1.1	(± 0.4)
Doctor/Clinical Officer	627	55.1%
Nurse/Midwife	594	52.2%
Other birth attendant	78	6.9%
No birth attendant	12	1.1%

* Percentages do not sum to 100% because women could report multiple support persons, timings of support, and birth attendants

### PC-ISP preferences and experiences

Most women indicated that they preferred having support persons integrated into their care. The average PC-ISP preference score was 3.47 (SD ± 0.91) out of a maximum of 4 (Table 3). Most women reported that they preferred integrating support persons, ranging from 73.6 to 93.6% for individual indicators. The highest proportion of women (93.6%) reported that they wanted their support persons to understand their condition/care and the fewest (73.6%) reported that they wanted to consult their support persons about care decisions.

Average PC-ISP experience score was 2.63 (SD ± 1.24) out of a maximum of 4 on the composite score. Most women reported positive experiences of PC-ISP: 77.9% reported that families felt welcome, 58.9% reported being given the opportunity to consult family on decisions, 76.7% reported that families were welcome to ask questions and 77.5% reported that providers listened to their family’s concerns. Only 45.3%, however, reported being asked by providers if their families should be told about their condition and/or care.

**Table 3:** Frequencies of PC-ISP preferences and experiences

**Women’s PC-ISP Preferences**	N or mean	% or (SD)
*Summative PC-ISP preference score (range 0–4)*	3.47	(± 0.91)
*Consult decisions*		
I wanted to consult my family about decisions about my birth care		
Agree	809	71.1%
Somewhat agree	28	2.5%
Disagree	301	26.4%
*Know condition/care*		
I wanted my family to know about my condition/care		
Agree	989	86.9%
Somewhat agree	24	2.1%
Disagree	125	11.0%
*Understand condition/care*		
I wanted my family to understand about my condition/care		
Agree	1,044	91.7%
Somewhat agree	21	1.9%
Disagree	73	6.4%
*Respects values*		
I would have liked my family members to make sure my provider respects my values and choices		
Agree	998	87.7%
Somewhat agree	35	3.1%
Disagree	105	9.2%
**Women’s PC-ISP Experiences**	**N or mean**	**% or (SD)**
*Summative PC-ISP experience score (range 0–4, excluding Felt Welcome)*	2.63	(± 1.24)
*Felt welcome*		
My family member(s) felt welcome by the facility during my birth		
Agree	857	75.3%
Somewhat Agree	30	2.6%
Disagree	242	21.3%
Don’t know	9	0.8%
*Opportunity to consult*		
I was given the opportunity by my health provider to consult my family about my health care decisions		
Agree	647	56.9%
Somewhat agree	23	2.0%
Disagree	468	41.1%
*Told condition/care*		
I was asked by my health provider if my family should be told about my condition/care		
Agree	499	43.9%
Somewhat agree	17	1.4%
Disagree	622	54.7%
*Welcome to ask questions* ^*1*^		
My family was welcome to ask my health care provider questions		
Agree	841	73.9%
Somewhat agree	32	2.8%
Disagree	216	19.0%
Don’t know	30	2.6%
N/A	19	1.7%
*Listened to concerns* ^*1*^		
My health care provider listened to my family members’ concerns		
Agree	839	73.7%
Somewhat agree	43	3.8%
Disagree	202	17.8%
Don’t know	30	2.6%
N/A	24	2.1%

^1^ N/A responses were excluded from analyses of individual items

### Factors associated with women’s PC-ISP preferences

Multivariable linear regression results showed that factors at multiple levels were associated with PC-ISP preferences. For the composite PC-ISP preference score, being married or partnered (*B* 0.18; 95% CI 0.00, 0.35), employed (*B* 0.12; 95% CI 0.01, 0.22), having a mother support person (*B* 0.35; 95% CI 0.10, 0.61), having postpartum support (*B* 0.12; 95% CI 0.05, 0.19) and being empowered in household decisions (*B* 0.11; 95% CI 0.03, 0.19) were associated with increased PC-ISP preference scores (Table 4). Women who had a mother support person had on average a 0.35 (95% CI 0.10, 0.61) higher PC-ISP preference score compared to those without a mother support person. In contrast, higher age (*B* -0.04; 95% CI -0.06, -0.01) and more support persons (*B*-0.18; 95% CI -0.29, -0.07) were negatively associated with the PC-ISP preferences score.

These results are consistent with logistic regression results for individual indicators, except for some associations with support person types and facility types (Appendix A). Individual PC-ISP preference indicators were inconsistently associated with facility types. Women in health centres were more likely to want to *consult* support persons for care decisions (aOR 1.25; 95% CI 1.10, 1.42), but less likely that support persons ensured that providers *respected their choices* (aOR 0.66; 95% CI 0.49, 0.88) compared to those in public hospitals. Women in private hospitals were less likely to want support persons to *know their condition/care* (aOR 0.76; 95% CI 0.59, 0.97) and to ensure providers *respected their choices* (aOR 0.51; 95% CI 0.34, 0.76).

For individual PC-ISP preference indicators and combined scores, separate examination of facilities did not reveal notable differences. Likelihood ratio tests of multi-level models did not find significant facility random effects, indicating that there were no systematic differences by facility.

**Table 4:** Factors associated with summative PC-ISP preference scores (multivariable linear regression model)

	Combined preference scores	
	*B*	95%CI
Age	-0.04**	(0.06, -0.01)
Parity	0.06	(-0.04, 0.15)
Marital status (Ref. Not married/partnered)		
Married or partnered	0.18*	(0.00, 0.35)
Education (ref. Primary or less)		
Vocational/Secondary	-0.06	(0.17, 0.06)
College/University	-0.06	(-0.40, 0.27)
Employment (ref. no)		
Yes	0.12*	(0.01, 0.22)
Birthplace (ref. born elsewhere)		
Born in Nairobi or Kiambu Counties	0.05	(-0.14, 0.24)
Self-rated health	-0.03	(-0.09, 0.03)
Covered under health scheme or health insurance (ref. No)		
Yes	0.04	(-0.21, 0.30)
Total support persons	-0.18*	(-0.29, -0.07)
Support person types		
Male partner (Ref. No)	0.21	(-0.02, 0.44)
Yes		
Mother (Ref. No)	0.35*	(0.10, 0.61)
Yes		
Mother-in-law (Ref. No)	0.26	(-0.23, 0.74)
Yes		
Father (Ref. No)	0.17	(-0.24, 0.58)
Yes		
Sister (Ref. No)	0.23	(-0.07, 0.52)
Yes		
Brother (Ref. No)	0.10	(-0.32, 0.53)
Yes		
Other family members (Ref. No)	0.21	(0.00, 0.43)
Yes		
Accompanied to facility (Ref. No support person accompanied)		
Yes	0.23	(-0.06, 0.52)
Labor & childbirth (Ref. No support person during L&D)		
Yes	0.09	(-0.13, 0.30)
Postpartum (Ref. No support person postpartum)		
Yes	0.12**	(0.05, 0.19)
Household decision-making (Ref. no say in all decisions)		
Yes	0.11*	(0.03, 0.19)
Facility type (Ref. Public hospital)		
Public health center	0.01	(-0.11, 0.13)
Private facility	-0.10	(-0.22, 0.01)
Total providers attending birth	-0.11	(-0.30, 0.08)
Selected facility based on quality	0.05	(-0.17, 0.26)
Referred to facility	0.14	(-0.02, 0.30)

Notes: *p < 0.05, **p < 0.01, ***p < 0.001 ; B = Beta coefficient

The Friend/Neighbor/Other support person measure was omitted from models because of collinearity

### Factors associated with women’s PC-ISP experiences

For linear regression results for the combined PC-ISP experiences scores, only one factor—having a male partner—was associated with an increase in total PC-ISP experience score (Table 5). Women who reported having a male partner support person, had 0.13-point higher PC-ISP experience scores compared to women without a male partner support person (*B* 0.13; 95% CI 0.02, 0.23).

Sensitivity analyses using a random intercept for facilities are presented in Appendix B. Increases in PC-ISP experience scores were associated with increased number of providers attending birth (*B* 0.29; 95% CI 0.10, 0.47) and being referred to a facility (*B*0.29; 95% CI 0.07, 0.52). Although the likelihood ratio test indicated a better fit for the random-intercept model than simple linear regression (p = 0.005), penalized measures of fit, Akaike Information Criterion (AIC) and Bayesian Information Criterion (BIC), indicated the simple linear regression model as a more parsimonious and better fit [40].

**Table 5:** Factors associated with total PC-ISP experience scores (n = 1,138)

	Combined experience scores	
	*B*	95% CI
Age	0.00	(-0.03, 0.02)
Parity	0.03	(-0.06, 0.13)
Marital status (Ref. Not married/partnered)		
Married or partnered	-0.09	(-0.34, 0.16)
Education (ref. Primary or less)		
Vocational/Secondary	0.00	(-0.17, 0.17)
College/University	-0.08	(-0.28, 0.13)
Employed (ref. no)		
Yes	0.07	(-0.18, 0.32)
Birthplace (ref. born elsewhere)		
Born in Nairobi or Kiambu Counties	-0.08	(-0.37, 0.21)
Self-rated health	-0.05	(-0.19, 0.10)
Covered under health scheme or health insurance (ref. No)		
Yes	0.06	(-0.09, 0.22)
Total support persons	-0.09	(-0.34, 0.16)
Male partner support person (Ref. No)		
Yes	0.13*	(0.02, 0.23)
Mother support person (Ref. No)		
Yes	0.12	(-0.12, 0.37)
Mother-in-law support person (Ref. No)		
Yes	-0.01	(-0.40, 0.39)
Father support person (Ref. No)		
Yes	0.14	(-0.94, 1.21)
Sister support person (Ref. No)		
Yes	0.01	(-0.29, 0.32)
Brother support person (Ref. No)		
Yes	0.12	(-0.39, 0.64)
Other family members support person (Ref. No)		
Yes	0.14	(-0.03, 0.31)
Timing of support: Accompanied to facility (Ref. No one accompanied)		
Yes	-0.04	(-0.48, 0.40)
Timing of support: Labor & childbirth (Ref. No one during L&C)		
Yes	0.03	(-0.15, 0.21)
Timing of support: Postpartum (Ref. No one postpartum)		
Yes	-0.04	(0.22, 0.13)
Household decision-making (Ref. Does not have say in all decisions)		
Yes	0.01	(-0.28, 0.30)
Facility type (Ref. Public hospital)		
Public health centre	0.16	(-0.12, 0.45)
Private facility	0.00	(-0.31, 0.30)
Total providers attending birth	0.29	(-0.01, 0.60)
Selected facility based on quality	0.15	(-0.07, 0.38)
Referred to facility	0.19	(-0.16, 0.54)

Notes: *p < 0.05, **p < 0.01, ***p < 0.001

The Friend/Neighbor/Other support person indicator was omitted from models because of collinearity

Logistic regression results of individual PC-ISP experience indicators showed that associations varied by factors at different levels (Appendix C). Employed women were more likely to report having the *opportunity to consult* support persons (aOR 1.25; 95% CI 1.07, 1.50) and providers asking if support persons should be *told about their condition/care* (aOR 1.29; 95% CI 1.07, 1.55). Having a greater number of support persons was associated with a lower likelihood of support persons being *welcome to ask questions* (aOR 0.71; 95% CI 0.53, 0.96) and providers *listening to support persons’ concerns* (aOR 0.77; 95% CI 0.62, 0.97). Women who reported having a male partner support person were more likely to report support persons *felt welcome* (aOR 1.64; 95% CI 1.41, 1.90). Postpartum support persons were more likely to be *welcome to ask questions* (aOR 1.34; 95% CI 1.04, 1.73). Women with a greater number of providers attending birth were more likely to report having the *opportunity to consult* support persons on decisions (aOR 1.53; 95% CI 1.09, 2.15) and support persons being welcome to *ask questions* (aOR 1.84; 95% CI 1.07, 2.54). Compared to public hospitals, women in public health centres were more likely to report an *opportunity to consult* support persons on decisions (aOR 1.42; 95% CI 1.27, 1.59) and providers asking if support persons should be *told about their condition/care* (aOR 1.30; 95% CI 1.08, 1.56). Women in private facilities were more likely to report support persons *felt welcome* (aOR 1.64; 95% CI 1.32, 2.03), but were less likely to be asked if support persons should be *told about their condition/care* (aOR 0.76; 95% CI 0.61, 0.94) compared to public hospitals.

## Discussion

We found an unmet need for support during childbirth. Although 30% wanted a support person during labor and/or childbirth, only 7.4% of women reported having one. Also, a large majority of women wanted their support persons integrated into their maternity care, but fewer women had them actually integrated in practice. Women and their support persons were treated differently due to differences in social status, support person types and facility characteristics. Further, women desired specific types of support aside from birth companionship.

### Women’s preferences for PC-ISP

Most women wanted their support persons to understand their condition/care and have their support persons make sure their providers respected their values and choices, but fewer wanted them to know about their condition/care or consult them on decisions, suggesting a desire to remain at the center of their own maternity care. Although there has been progress, women’s participation in health care is still low, especially in low and middle-income countries, and these findings highlight the need to increase women’s involvement in their maternity care [34].

Consistent with findings from other studies in Kenya, younger women preferred integrating support persons, which is likely because they are less experienced with the birthing process and need an advocate during care [8]. Similarly, our findings suggest women are more likely to look to their mothers for multiple supportive functions. Further, the increased likelihood of wanting to consult for decisions with mothers, male partners, sisters and other family members implies maternity care needs to expand access to a variety of other support persons. Literature and health policies have predominantly focused on male partner involvement and providers frequently give preferential treatment to male partners [8, 33, 41–44]. Yet, many studies have found preferences for mothers and other female relatives, often stemming from cultural preferences [12, 22, 45, 46]. Maternity care systems must thus broaden efforts to facilitate support persons’ involvement beyond male partners.

Interestingly, more support persons were negatively associated with several PC-ISP preference indicators. Women may need more privacy if multiple support persons are involved. They may consider certain individuals to coach them they find unhelpful and want to limit some individuals’ involvement [47, 48].

Women who have a say among their household members, also are more likely to integrate them into their care. When you, however, lack decision-making power at home, you may want to protect your autonomy in health care and not prefer to integrate support persons in your care [29]. Women’s decision-making power in health care is increasing in sub-Saharan Africa, suggesting that women may increasingly prefer to integrate support persons [49].

### Women’s experiences of PC-ISP

Fewer women reported experiences of PC-ISP compared to those who preferred it, underscoring how the healthcare system is ill-equipped to facilitate support. Maternity care providers lack a clear agenda for engaging support persons directly or productively [33]. Providers often have widely differing practices about engaging support persons. They may only allow certain types of support persons or only provide information about women’s conditions if women experience problems [9, 33].

More attention is needed on the equitable, inclusive treatment of support persons, since women may need a variety of support persons. Few studies, however, have investigated strategies to address discriminatory or differential treatment of support persons and intervention studies that aim to increase access to support persons, reported differential treatment [31]. Future research should assess whether they are treated differently in practice and how this may affect women’s receipt of support.

Women’s needs for support are greatest during labor, childbirth and postpartum, so excluding them then would probably be the most hazardous [50, 51]. Allowing women their support persons throughout maternity care contributes to their network members’ sense of inclusion and belonging in care [24]. Helping families and social network members feel welcome in facilities influences positive perceptions of facility-based care in the broader community, which contributes to women’s and community members’ future decisions to utilize health care [4, 6, 26, 52].

Our study corroborates existing literature that facility-level factors influence women’s access to support persons [7, 8, 11]. Adequate staffing contributes to providers’ capacity to engage with support persons and facilitate support. Providers had less capacity to engage as the number of support persons increased. Other studies suggested exclusion of support persons arise from understaffed wards and overworked providers [8, 11, 31, 53, 54]. Women’s access to support persons largely depended upon facilities’ “*allocation of resources, organization of care, facility-related constraints and cultural inclinations.”* [7]. These institutional factors also highlight how health care providers are embedded in institutions, health care and social systems. Despite aiming to provide the best possible care, providers are often constrained and frustrated by institutional policies, lack of resources and social hierarchies [55–57]. Future research should further estimate how additional facility factors, such as financial resources or physical infrastructure, are associated with women’s access to support persons. This information is critical for developing comprehensive, multi-level interventions to increase support for women [20].

### Limitations and Future directions

This study makes an important contribution to the literature on how support persons fit into a person-centered maternity care approach, but there are notable limitations. The PC-ISP survey measures were developed and applied specifically for intrapartum care and do not reflect preferences and experiences of care during other periods (e.g., antenatal, postpartum). PC-ISP measures were not validated within the sample, nor did they undergo psychometric or formal scale development. Additionally, given low reliability, the combined PC-ISP scores may not accurately or validly measure PC-ISP as a broader concept. Past studies, however, have been primarily qualitative or only used single measures that do not adequately capture the PC-ISP construct. This study initiates research on measuring integration of support persons and the proposed subconstructs merit further examination in other contexts.

Although women were surveyed about their PC-ISP preferences and experiences, we were unable to assess how preferences aligned with or influenced reports of their PC-ISP experiences. Because acknowledging and respecting women’s preferences is important for person-centered care, knowledge of women’s preferences for integrating support persons in maternity care is an important area of needed research. More research is also needed to assess the extent to which women’s PC-ISP preferences affect their experiences of care and its quality.

Another limitation was that women were interviewed only once during the postpartum period. Because women reported their preferences after they received care, responses were possibly influenced by their experiences of care. The analysis also limits our ability to draw causal conclusions about factors associated with PC-ISP because of the number of factors included in final models, potentially because some may have reached significance by chance. Finally, there is limited generalizability, because our sample included only women who gave birth in six facilities in urban areas in Nairobi and Kiambu counties. Moreover, there was notable heterogeneity in facility characteristics, for example, of the two private hospitals, one was for-profit and one was not-for-profit. More research is needed across a greater number of facilities to better examine women’s access to support persons and how facility factors influence PC-ISP. Nevertheless, this study generates insights about gaps in maternity care and how health systems may better integrate support persons to improve person-centered care.

### Conclusions and implications

Childbirth constitutes a time when women are at high-risk for mortality, morbidity and mistreatment. Because support persons play critical functions and are important for women, efforts to improve person-centered care should also include integrating support persons as women need them. Integrating support persons can bolster women’s autonomy and involvement in their own care [10, 27]. Maternity care needs appropriate instruments to better assess women’s preferences for support and the measures in this study could inform future efforts to develop validated tools for the PC-ISP construct. The health care system should also establish processes to incorporate women’s preferences to guide maternity care. At a minimum, the care team should acknowledge and engage with women’s support persons of choice. Training for providers is needed to ensure equitable treatment of women and allow different types of support persons as women rely on different types for different forms of support. Lastly, efforts to integrate support persons into maternity care need to address facility factors including staff capacity, cultures of care that rationalize mistreatment and patterns of providers’ negative attitudes and disrespect towards support persons [8, 23]. Providers need training about the benefits of integrating support persons for women’s care and community health workers may be able to fill in gaps in staff shortages for non-technical care. Ultimately, integrating support persons into maternity care as women need them, bolsters efforts to advance person-centered care and has the potential to improve health outcomes [10, 58].

### Electronic supplementary material

Below is the link to the electronic supplementary material.

Supplementary Material 1: : Appendix A

Supplementary Material 2: : Appendix B

Supplementary Material 3: : Appendix C

## Data Availability

data that support the findings of this study are available from the corresponding author upon reasonable request.

## Declarations

All methods were performed in accordance with the Declaration of Helsinki. Informed consent was obtained from all participants prior to participation. Consent from women aged 15–17 was obtained equally as from those above aged 18 because they were considered mature minors (defined as those who are married, a mother, pregnant or a household head) and informed consent from legal guardians/parents was waived by both IRBs.
