# Supplementary material for: Access to support during childbirth?: women’s preferences and experiences of support person integration in a cross-sectional facility-based survey
Source: BMC Pregnancy Childbirth. 2023 Sep 16;23:665. doi: 10.1186/s12884-023-05962-2 (PMC10504704; doi:10.1186/s12884-023-05962-2)
Supplement: Supplementary file 1 — Supplementary Material 1: : Appendix A [file 12884_2023_5962_MOESM1_ESM.docx]

**Appendix A.** Multivariable logistic models for separate PC-ISP preference indicators

|  | ***Consult decisions*** | | | ***Know condition/care*** | | | ***Understand condition/care*** | | | | | | ***Respect choice*** | | | | | |  |
| --- | --- | --- | --- | --- | --- | --- | --- | --- | --- | --- | --- | --- | --- | --- | --- | --- | --- | --- | --- |
|  | **aOR** | **95%CI** | | **aOR** | **95%CI** | | | **aOR** | | **95%CI** | | | | **aOR** | | **95%CI** | | | |
| Age | 0.93** | (0.88, | 0.98) | 0.91*** | (0.88, | 0.93) | 0.90*** | | (0.88, | | 0.92) | 0.93*** | | | (0.91, | | 0.96) |  |  |
| Parity | 1.09 | (0.92, | 1.30) | 1.26* | (1.03, | 1.54) | 1.34* | | (1.00, | | 1.80) | 0.95 | | | (0.76, | | 1.19) |  |  |
| Marital status (Ref. Not married/partnered) |  |  |  |  |  |  |  | |  | |  |  | | |  | |  |  |  |
| Married or partnered | 1.15 | (0.79, | 1.67) | 1.93* | (1.11, | 3.35) | 3.25* | | (1.14, | | 9.30) | 1.45 | | | (0.67, | | 3.12) |  |  |
| Education (ref. Primary or less) |  |  |  |  |  |  |  | |  | |  |  | | |  | |  |  |  |
| Vocational/Secondary | 0.71** | (0.58, | 0.88) | 0.94 | (0.63, | 1.39) | 1.14 | | (0.79, | | 1.65) | 0.97 | | | (0.71, | | 1.33) |  |  |
| College/University | 0.68 | (0.37, | 1.24) | 1.03 | (0.54, | 1.96) | 0.82 | | (0.33, | | 2.03) | 1.10 | | | (0.54, | | 2.24) |  |  |
| Employed (ref. no) |  |  |  |  |  |  |  | |  | |  |  | | |  | |  |  |  |
| Yes | 1.26 | (0.86, | 1.85) | 1.28* | (1.00, | 1.64) | 1.53 | | (0.89, | | 2.64) | 1.33 | | | (0.84, | | 2.11) |  |  |
| Birthplace (ref. born elsewhere) |  |  |  |  |  |  |  | |  | |  |  | | |  | |  |  |  |
| Born in Nairobi or Kiambu Counties | 0.79 | (0.60, | 1.05) | 1.30 | (0.61, | 2.77) | 1.53 | | (0.53, | | 4.39) | 1.96** | | | (1.18, | | 3.26) |  |  |
| Self-rated health | 0.95 | (0.80, | 1.13) | 0.94 | (0.79, | 1.13) | 0.98 | | (0.84, | | 1.14) | 0.89 | | | (0.78, | | 1.02) |  |  |
| Covered under health scheme or health insurance (ref. No) |  |  |  |  |  |  |  | |  | |  |  | | |  | |  |  |  |
| Yes | 1.14 | (0.89, | 1.46) | 1.01 | (0.45, | 2.25) | 0.87 | | (0.29, | | 2.62) | 1.33 | | | (0.61, | | 2.88) |  |  |
| Total number of support persons | 0.67* | (0.48, | 0.94) | 0.65*** | (0.51, | 0.83) | 0.53*** | | (0.36, | | 0.76) | 0.80 | | | (0.56, | | 1.14) |  |  |
| Support person types |  |  |  |  |  |  |  | |  | |  |  | | |  | |  |  |  |
| Male partner (Ref. No) |  |  |  |  |  |  |  | |  | |  |  | | |  | |  |  |  |
| Yes | 1.98*** | (1.51, | 2.61) | 1.34 | (0.74, | 2.43) | 1.12 | | (0.63, | | 2.02) | 1.46 | | | (0.95, | | 2.23) |  |  |
| Mother (Ref. No) |  |  |  |  |  |  |  | |  | |  |  | | |  | |  |  |  |
| Yes | 3.67*** | (1.74, | 7.73) | 2.45 | (0.76, | 7.86) | 9.92*** | | (3.01, | | 32.66) | 0.88 | | | (0.40, | | 1.93) |  |  |
| Mother-in-law (Ref. No) |  |  |  |  |  |  |  | |  | |  |  | | |  | |  |  |  |
| Yes | 1.57 | (0.46, | 5.37) | 2.38 | (0.38, | 14.92) | 3.24 | | (0.23, | | 46.31) | 1.69 | | | (0.64, | | 4.45) |  |  |
| Father (Ref. No) |  |  |  |  |  |  |  | |  | |  |  | | |  | |  |  |  |
| Yes | 1.53 | (0.62, | 3.77) | 0.44* | (0.19, | 0.99) |  | |  | |  |  | | |  | |  |  |  |
| Sister (Ref. No) |  |  |  |  |  |  |  | |  | |  |  | | |  | |  |  |  |
| Yes | 1.77* | (1.09, | 2.88) | 1.43 | (0.57, | 3.60) | 1.86 | | (0.65, | | 5.36) | 1.64 | | | (0.83, | | 3.25) |  |  |
| Brother (Ref. No) |  |  |  |  |  |  |  | |  | |  |  | | |  | |  |  |  |
| Yes | 0.82 | (0.40, | 1.67) | 2.87 | (0.23, | 35.37) | 1.80 | | (0.11, | | 30.65) | 1.62 | | | (0.16, | | 16.09) |  |  |
| Other family members (Ref. No) |  |  |  |  |  |  |  | |  | |  |  | | |  | |  |  |  |
| Yes | 1.55* | (1.02, | 2.35) | 1.91* | (1.04, | 3.48) | 2.65** | | (1.42, | | 4.91) | 1.20 | | | (0.84, | | 1.71) |  |  |
| Accompanied to facility (Ref. No support person accompanied) |  |  |  |  |  |  |  | |  | |  |  | | |  | |  |  |  |
| Support person accompanied | 1.44* | (1.01, | 2.06) | 2.47 | (1.41, | 4.34) | 2.24 | | (0.80, | | 6.29) | 1.05 | | | (0.84, | | 1.31) |  |  |
| Labor & Childbirth Support (Ref. No one during L&C) |  |  |  |  |  |  |  | |  | |  |  | | |  | |  |  |  |
| Had support person during Labor and/or Childbirth | 1.27 | (0.96, | 1.69) | 1.22 | (0.43, | 3.43) | 1.49 | | (0.41, | | 5.35) | 1.08 | | | (0.69, | | 1.70) |  |  |
| Postpartum (Ref. No one postpartum) |  |  |  |  |  |  |  | |  | |  |  | | |  | |  |  |  |
| Had support person during Postpartum | 1.26 | (0.94, | 1.69) | 1.76*** | (1.29, | 2.41) | 1.74 | | (0.95, | | 3.21) | 0.92 | | | (0.64, | | 1.32) |  |  |
| Household decision-making (Ref. Does not have say in all decisions) |  |  |  |  |  |  |  | |  | |  |  | | |  | |  |  |  |
| Empowered in HH decisions | 1.26** | (1.08, | 1.47) | 1.53** | (1.15, | 2.03) | 1.65** | | (1.13, | | 2.41) | 0.94 | | | (0.60, | | 1.47) |  |  |
| Facility type (Ref. Public hospital) |  |  |  |  |  |  |  | |  | |  |  | | |  | |  |  |  |
| Public HC/Disp | 1.25*** | (1.10, | 1.42) | 1.18 | (0.86, | 1.62) | 0.85 | | (0.45, | | 1.60) | 0.66** | | | (0.49, | | 0.88) |  |  |
| Private facility | 0.89 | (0.79, | 1.01) | 0.76* | (0.59, | 0.97) | 1.09 | | (0.57, | | 2.09) | 0.51** | | | (0.34, | | 0.76) |  |  |
| Total providers attending birth | 0.84 | (0.60, | 1.17) | 0.78 | (0.56, | 1.09) | 0.84 | | (0.50, | | 1.40) | 0.65 | | | (0.38, | | 1.11) |  |  |
| Selected facility based on quality | 1.22 | (0.90, | 1.65) | 0.71 | (0.42, | 1.22) | 1.17 | | (0.67, | | 2.01) | 1.33 | | | (0.67, | | 2.64) |  |  |
| Referred to facility | 1.62*** | (1.25, | 2.10) | 1.08 | (0.84, | 1.39) | 1.56 | | (0.84, | | 2.90) | 1.25 | | | (0.55, | | 2.83) |  |  |

Notes: *p<0.05, **p<0.01, ***p<0.001
