# Supplementary material for: Access to support during childbirth?: women’s preferences and experiences of support person integration in a cross-sectional facility-based survey
Source: BMC Pregnancy Childbirth. 2023 Sep 16;23:665. doi: 10.1186/s12884-023-05962-2 (PMC10504704; doi:10.1186/s12884-023-05962-2)
Supplement: Supplementary file 2 — Supplementary Material 2: : Appendix B [file 12884_2023_5962_MOESM2_ESM.docx]

**Appendix B.** Factors associated with total PC-ISP experience score, random intercept model

|  | **Combined experiences score** | |
| --- | --- | --- |
|  | ***B*** | **95% CI** |
| Age | 0.00 | (-0.02, 0.02) |
| Parity | 0.02 | (-0.09, 0.13) |
| Marital status (Ref. Not married/partnered) |  |  |
| Married or partnered | -0.10 | (-0.35, 0.14) |
| Education (ref. Primary or less) |  |  |
| Vocational/Secondary | -0.02 | (-0.19, 0.14) |
| College/University | -0.10 | (-0.33, 0.13) |
| Employed (ref. no) |  |  |
| Yes | 0.07 | (-0.08, 0.23) |
| Birthplace (ref. born elsewhere) |  |  |
| Born in Nairobi or Kiambu Counties | -0.10 | (-0.29, 0.09) |
| Self-rated health | -0.05 | (-0.12, 0.03) |
| Covered under health scheme or health insurance (ref. No) |  |  |
| Yes | 0.09 | (-0.11, 0.30) |
| Total support persons | -0.09 | (-0.26, 0.08) |
| Support person types |  |  |
| Male partner (Ref. No) |  |  |
| Yes | 0.13 | (-0.07, 0.33) |
| Mother (Ref. No) |  |  |
| Yes | 0.11 | (-0.20, 0.42) |
| Mother-in-law (Ref. No) |  |  |
| Yes | -0.03 | (-0.46, 0.41) |
| Father (Ref. No) |  |  |
| Yes | 0.11 | (-0.66, 0.89) |
| Sister (Ref. No) |  |  |
| Yes | 0.02 | (-0.21, 0.25) |
| Brother (Ref. No) |  |  |
| Yes | 0.18 | (-0.41, 0.78) |
| Other family members (Ref. No) |  |  |
| Yes | 0.15 | (-0.07, 0.36) |
| Timing of support: Accompanied to facility (Ref. No one accompanied) |  |  |
| Accompanied | -0.01 | (-0.33, 0.30) |
| Timing of support: Labor & childbirth (Ref. No one during L&C) |  |  |
| Labor & Childbirth | 0.01 | (-0.26, 0.28) |
| Timing of support: Postpartum (Ref. No one postpartum) |  |  |
| Postpartum | -0.05 | (-0.21, 0.10) |
| Household decision-making (Ref. No say in all decisions) |  |  |
| Empowered in HH decisions | 0.02 | (-0.20, 0.16) |
| Facility type (Ref. Public hospital) |  |  |
| Public HC/Disp | 0.16 | (-0.30, 0.53) |
| Private facility | 0.01 | (-0.30, 0.32) |
| Total providers attending birth | 0.29** | (0.10, 0.47) |
| Selected facility based on quality | 0.14 | (-0.03, 0.30) |
| Referred to facility | 0.29* | (0.07, 0.52) |
| Random effects at the facility-level |  | SE |
| σ_u_^2^ | 0.0156 | 0.0135 |
| $\hat{\sigma}_{\varepsilon}^{2}$ | 1.453 | 0.0611 |

Notes: *p<0.05, **p<0.01, ***p<0.001

The Friend/Neighbor/Other support person indicator was omitted from models because of collinearity.
