# Supplementary material for: Access to support during childbirth?: women’s preferences and experiences of support person integration in a cross-sectional facility-based survey
Source: BMC Pregnancy Childbirth. 2023 Sep 16;23:665. doi: 10.1186/s12884-023-05962-2 (PMC10504704; doi:10.1186/s12884-023-05962-2)
Supplement: Supplementary file 3 — Supplementary Material 3: : Appendix C [file 12884_2023_5962_MOESM3_ESM.docx]

**Appendix C.** Multivariable logistic regression models for separate PC-ISP experience indicators

|  | ***Felt welcome*** | | ***Opportunity to consult*** | | ***Told condition/care*** | | ***Welcome to ask questions*** | | ***Listened to concerns*** | |
| --- | --- | --- | --- | --- | --- | --- | --- | --- | --- | --- |
|  | **aOR** | **95% CI** | **aOR** | **95% CI** | **aOR** | **95% CI** | **aOR** | **95% CI** | **aOR** | **95% CI** |
| Age | 0.98** | (0.96, 0.99) | 0.99 | (0.94, 1.03) | 1.00 | (0.97, 1.02) | 1.00 | (0.98, 1.03) | 1.01 | (0.97, 1.05) |
| Parity | 1.00 | (0.83, 1.20) | 1.13* | (1.02, 1.24) | 1.02 | (0.90, 1.15) | 0.99 | (0.83, 1.17) | 1.09 | (0.90, 1.31) |
| Marital status (Ref. Not married/partnered) |  |  |  |  |  |  |  |  |  |  |
| Married or partnered | 0.72 | (0.45, 1.14) | 1.00 | (0.65, 1.53) | 0.83* | (0.69, 1.00) | 0.96 | (0.76, 1.22) | 0.86 | (0.73, 1.02) |
| Education (ref. Primary or less) |  |  |  |  |  |  |  |  |  |  |
| Vocational/Secondary | 0.91 | (0.78, 1.06) | 0.92 | (0.70, 1.19) | 1.07 | (0.73, 1.57) | 0.92 | (0.71, 1.19) | 1.17*** | (1.07, 1.28) |
| College/University | 0.91 | (0.51, 1.62) | 0.71 | (0.49, 1.04) | 0.82 | (0.47, 1.42) | 1.04 | (0.60, 1.82) | 1.51*** | (1.21, 1.89) |
| Employed (ref. no) |  |  |  |  |  |  |  |  |  |  |
| Yes | 1.05 | (0.71, 1.55) | 1.26** | (1.07, 1.50) | 1.29** | (1.07, 1.55) | 0.90 | (0.54, 1.51) | 0.78 | (0.50, 1.22) |
| Birthplace (ref. born elsewhere) |  |  |  |  |  |  |  |  |  |  |
| Born in Nairobi or Kiambu Counties | 1.29* | (1.04, 1.60) | 1.05 | (0.82, 1.36) | 0.91 | (0.57, 1.46) | 0.92 | (0.62, 1.37) | 0.75 | (0.43, 1.31) |
| Self-rated health | 0.93 | (0.83, 1.05) | 0.94 | (0.82, 1.09) | 0.89* | (0.83, 0.96) | 1.02 | (0.85, 1.22) | 0.99 | (0.78, 1.24) |
| Covered under health scheme or health insurance (ref. No) |  |  |  |  |  |  |  |  |  |  |
| Yes | 0.70* | (0.50, 0.97) | 1.24 | (0.95, 1.61) | 1.19 | (0.93, 1.52) | 0.98 | (0.85, 1.13) | 1.00 | (0.78, 1.24) |
| Total support persons | 0.99 | (0.69, 1.41) | 0.94 | (0.67, 1.32) | 1.00 | (0.70, 1.42) | 0.71* | (0.53, 0.96) | 0.77* | (0.62, 0.97) |
| Support person types |  |  |  |  |  |  |  |  |  |  |
| Male partner (Ref. No) |  |  |  |  |  |  |  |  |  |  |
| Yes | 1.64*** | (1.41, 1.90) | 1.11 | (0.90, 1.37) | 1.11 | (0.91, 1.35) | 1.15 | (0.85, 1.55) | 1.47* | (1.07, 2.03) |
| Mother (Ref. No) |  |  |  |  |  |  |  |  |  |  |
| Yes | 1.36 | (0.84, 2.21) | 1.50*** | (1.20, 1.86) | 1.23 | (0.78, 1.95) | 0.91 | (0.53, 1.55) | 0.91 | (0.41, 2.05) |
| Mother-in-law (Ref. No) |  |  |  |  |  |  |  |  |  |  |
| Yes | 1.24 | (0.57, 2.72) | 1.24 | (0.72, 2.13) | 0.86 | (0.40, 1.83) | 0.76 | (0.56, 1.03) | 1.14 | (0.60, 2.15) |
| Father (Ref. No) |  |  |  |  |  |  |  |  |  |  |
| Yes | 0.17*** | (0.09, 0.33) | 1.29 | (0.20, 8.46) | 0.34 | (0.11, 1.12) | 2.95 | (0.55, 15.77) | 3.14 | (0.69, 14.29) |
| Sister (Ref. No) |  |  |  |  |  |  |  |  |  |  |
| Yes | 0.99 | (0.62, 1.59) | 1.17 | (0.85, 1.61) | 1.00 | (0.76, 1.32) | 0.81 | (0.48, 1.38) | 1.07 | (0.73, 1.58) |
| Brother (Ref. No) |  |  |  |  |  |  |  |  |  |  |
| Yes | 0.47* | (0.24, 0.92) | 0.77 | (0.58, 1.03) | 0.95 | (0.41, 2.19) | 1.47 | (0.36, 6.08) | 2.94 | (0.70, 12.24) |
| Other family members (Ref. No) |  |  |  |  |  |  |  |  |  |  |
| Yes | 0.95 | (0.54, 1.67) | 1.16 | (1.00, 1.36) | 1.23 | (0.84, 1.80) | 1.21 | (0.98, 1.50) | 1.38 | (0.91, 2.09) |
| Accompanied to facility (Ref. No one accompanied) |  |  |  |  |  |  |  |  |  |  |
| Support person Accompanied | 0.85 | (0.55, 1.32) | 0.99 | (0.73, 1.35) | 0.74 | (0.37, 1.47) | 1.08 | (0.71, 1.62) | 0.86 | (0.54, 1.38) |
| Labor & Childbirth (Ref. No one during L&C) |  |  |  |  |  |  |  |  |  |  |
| Had support person during Labor & Childbirth | 2.34* | (1.16, 4.71) | 1.24 | (0.89, 1.75) | 1.01 | (0.74, 1.37) | 1.30 | (0.67, 1.62) | 0.79 | (0.45, 1.38) |
| Postpartum (Ref. No one postpartum) |  |  |  |  |  |  |  |  |  |  |
| Had support person Postpartum | 1.94** | (1.29, 2.94) | 0.81 | (0.56, 1.18) | 0.74*** | (0.67, 0.81) | 1.34* | (1.04, 1.73) | 1.14 | (0.73, 1.34) |
| Household decision-making (Ref. Does not have say in all decisions) |  |  |  |  |  |  |  |  |  |  |
| Empowered in HH decisions | 0.93 | (0.69, 1.25) | 1.00 | (0.74, 1.36) | 1.21 | (0.83, 1.76) | 0.92 | (0.79, 1.07) | 0.92 | (0.63, 1.96) |
| Facility type (Ref. Public hospital) |  |  |  |  |  |  |  |  |  |  |
| Public HC/Disp | 0.72*** | (0.63, 0.82) | 1.42*** | (1.27, 1.59) | 1.30** | (1.08, 1.56) | 1.09 | (0.69, 1.73) | 1.17 | (0.70, 1.96) |
| Private facility | 1.64*** | (1.32, 2.03) | 1.03 | (0.87, 1.22) | 0.76* | (0.61, 0.94) | 1.08 | (0.45, 1.62) | 1.18 | (0.66, 2.09) |
| Total providers attending birth | 0.85 | (0.51, 1.41) | 1.53* | (1.09, 2.15) | 1.10 | (0.78, 1.54) | 1.84* | (1.07, 2.54) | 1.64 | (0.87, 3.07) |
| Selected facility based on quality | 0.87 | (0.55, 1.38) | 1.18 | (0.95, 1.47) | 1.05 | (0.74, 1.51) | 1.21* | (1.01, 1.46) | 1.35* | (1.05, 1.73) |
| Referred to facility | 0.92 | (0.58, 1.45) | 1.42** | (1.09, 1.84) | 1.30 | (0.89, 1.90) | 1.06 | (0.76, 1.48) | 1.14 | (0.72, 1.79) |

Notes: *p<0.05, **p<0.01, ***p<0.001
